# Right ventricular dysfunction during induction of anesthesia in an infant with repaired truncus arteriosus: a case report and learning from a potentially preventable adverse perioperative event

**DOI:** 10.3389/fmed.2026.1960952

**Published:** 2026-10-22

**Authors:** Jing Sun, Wenqin Zhou

**Affiliations:** 1 Department of Anesthesiology Nursing, West China Second Hospital of Sichuan University, Chengdu, China; 2 Key Laboratory of Birth Deficits and Related Diseases of Women and Children, Sichuan University, Ministry of Education, Chengdu, China

**Keywords:** acute right ventricular dysfunction, anesthetic induction, epinephrine, systemic systolic blood pressure, truncus arteriosus, ventricular interdependence

## Abstract

**Background:**

Infants following repair of truncus arteriosus frequently present with right ventricular (RV) dilation and left ventricular (LV) compression, predisposing them to acute right heart failure during non-cardiac surgery. Anesthetic induction and tracheal intubation may further exacerbate hemodynamic instability. Judicious induction strategies and meticulous airway management are therefore essential in this vulnerable population.

**Case presentation:**

We report a 4-month-old infant with repaired truncus arteriosus who developed progressive bradycardia, hypotension, and systemic venous congestion approximately one minute after tracheal intubation. The bradycardia was unresponsive to atropine but was rapidly reversed by a low-dose intravenous bolus of epinephrine (1 μg/kg). A transient surge in end-tidal carbon dioxide (ETCO₂) from 30 to 70 mmHg immediately after epinephrine administration indicated restoration of right ventricular forward flow. Postoperative biomarkers were markedly elevated [B-type Natriuretic Peptide (BNP) 639.58 pg/mL, troponin 79.14 ng/L]. Drawing upon the framework of ventricular interdependence, we analyze the vicious cycle triggered by the combined effects of reduced systemic vascular resistance (SVR) and positive-pressure ventilation, and propose perioperative management strategies tailored to infants with a “large RV and compressed LV” phenotype. At one-month follow-up, the infant remained asymptomatic with stable echocardiographic parameters.

**Conclusions:**

This case suggests that infants with a “large RV, small LV” phenotype may be at increased risk for acute right ventricular dysfunction (RVD) during anesthesia. Multiple factors—including pre-existing RVD, induction anesthetics, positive-pressure ventilation, and lung overinflation—may have collectively contributed to the event. Maintaining systemic systolic pressure, avoiding excessive lung inflation, and having low-dose epinephrine readily available appear to be prudent measures in such patients. However, these observations are derived from a single case and should be interpreted with caution.

## 1 Introduction

Infants following repair of truncus arteriosus frequently have residual right ventricular outflow tract (RVOT) stenosis, pulmonary regurgitation, and pre-existing right heart dysfunction (1–3). This hemodynamic profile—characterized by a dilated RV and a compressed LV—renders these patients exceptionally vulnerable during the perioperative period.

Under normal physiology, the RV ejects blood into a low-resistance, high-compliance pulmonary circulation and is exquisitely sensitive to acute increases in afterload (4). When RV afterload rises abruptly, rapid RV dilation and leftward displacement of the interventricular septum occur, severely compromising LV filling through the mechanism of ventricular interdependence, culminating in circulatory collapse (5). Anesthetic-induced myocardial depression and vasodilation, compounded by elevated pulmonary vascular resistance (PVR) from positive-pressure ventilation, may collectively precipitate acute right heart decompensation in these vulnerable patients (6). While perioperative management strategies for pulmonary hypertension and RVD have been broadly discussed, few reports provide a detailed, real-time analysis of the pathophysiological sequence of events and a practical rescue protocol in infants with repaired truncus arteriosus. This case report offers three specific educational messages for the practicing anesthesiologist: (1) a cautionary example that even reduced-dose propofol can be unsafe in this population; (2) a systematic framework for differential diagnosis of acute intraoperative bradycardia with facial congestion; and (3) a practical rescue algorithm centered on low-dose epinephrine and maintenance of systemic systolic pressure.

## 2 Case presentation

### 2.1 Preoperative data

The patient was a 4-month and 16-day-old male infant, weighing 5 kg with a body length of 66 cm, admitted for left congenital talipes equinovarus, scheduled for Achilles tenotomy and corrective surgery. At 3 months of age, he underwent repair of truncus arteriosus (Van Praagh type A1) with ventricular septal defect (VSD) closure using a pericardial patch and RVOT reconstruction with a 14-mm pulmonary homograft. After weaning from cardiopulmonary bypass, transesophageal echocardiography (TEE) showed no residual VSD shunt and unobstructed RVOT flow [peak velocities: Main Pulmonary Artery (PA) 1.9 m/s, left PA 2.2 m/s, right PA 2.2 m/s]. Intraoperative pressure measurement revealed a right ventricular systolic pressure (RVSP)/left ventricular systolic pressure (LVSP) ratio of 0.4 (34/80 mmHg). Due to hemodynamic instability requiring high vasoactive support, delayed sternal closure and temporary epicardial pacing wires were placed. Pacing status during TEE was not documented.

Transthoracic echocardiography performed one month post-cardiac surgery revealed the following: no significant residual ventricular septal shunt; accelerated antegrade pulmonary blood flow; pulmonary regurgitation; mild aortic regurgitation; mild tricuspid regurgitation; normal LV systolic function [ejection fraction [EF] = 62%, fractional shortening [FS] = 31%]; An increased right ventricular proportion with a disproportionately large right atrium; a compressed small LV cavity [left atrium [LA] = 15 mm, LV = 17 mm, right atrium [RA] = 22 × 24 mm, RV = 15 mm]; a narrowest RVOT diameter of 7.3 mm; accelerated pulmonary artery flow with a peak velocity (Vmax) of 3.3 m/s; and tricuspid regurgitation with a Vmax of 3.8 m/s and an estimated pressure gradient of 58 mmHg, corresponding to an estimated RVSP of approximately 63 mmHg. These findings indicated chronic RV pressure overload.

The preoperative electrocardiogram showed sinus rhythm, right-axis deviation (+162°), complete right bundle branch block, and T-wave changes. Chest radiography suggested possible bilateral pulmonary inflammation, though the infant was afebrile, without cough, feeding normally, and exhibited no hypoxic spells, with clear breath sounds on auscultation.

### 2.2 Preoperative risk assessment and anesthetic planning

Before surgery, the cardiac surgeon was consulted prior to the orthopedic procedure and considered the patient's cardiac status to be stable enough to tolerate this short, minor surgery. Therefore, the operation was not delayed. A repeat echocardiogram was not performed immediately before the orthopedic procedure, which we acknowledge as a limitation of this case.

Based on the echocardiographic findings of severe RV enlargement, LV compression, and residual RVOT stenosis, we recognized this infant as being at high risk for acute RVD during anesthesia. Our preoperative plan prioritized: (1) maintenance of systemic systolic blood pressure to preserve RV coronary perfusion; (2) avoidance of drugs that depress myocardial contractility or reduce SVR; and (3) preparation of diluted epinephrine for immediate rescue. In retrospect, however, we failed to strictly adhere to this plan, which contributed to the subsequent critical event. In particular, the use of propofol (even low dose) reflected an underestimation of the infant's hemodynamic fragility, given his clinically stable preoperative appearance.

### 2.3 Anesthetic induction and critical event

Upon arrival in the operating room, monitoring showed heart rate (HR) 115 bpm, blood pressure (BP) 90/55 mmHg, and peripheral oxygen saturation (SpO₂) 100%. Anesthesia was induced with atropine 0.1 mg, midazolam 0.25 mg, cisatracurium 1 mg, propofol 5 mg (1 mg/kg), and 2% sevoflurane inhalation, followed by fentanyl 15 μg and an additional 10 μg after 3 min. Tracheal intubation was performed uneventfully. Post-intubation auscultation revealed clear and symmetrical breath sounds. After lung inflation, pressure-controlled ventilation was initiated with a driving pressure of 12 cmH_2_O, tidal volume 40 mL, respiratory rate 30 breaths/min, and initial ETCO₂ of 30 mmHg, HR 127 bpm, and BP 67/55 mmHg.

Approximately one minute after intubation, the infant developed progressive bradycardia, with HR declining from 120 to 95 bpm within one minute, and further to 60 bpm. Intravenous atropine 0.05 mg was administered without effect. Immediate rescue with intravenous epinephrine 5 μg (1 μg/kg) rapidly increased HR to 130 bpm and BP to 85/66 mmHg, and ETCO₂ rose abruptly from 30 to 70 mmHg within approximately 5 s. Simultaneously, the infant developed facial and cranial venous congestion with a purplish, blanchable discoloration. Facial congestion resolved within approximately 2 min, and ETCO₂ returned to 40 mmHg. Femoral arterial catheterization was performed. Arterial blood gas analysis at this point showed: pH 7.282, PCO₂ 53.3 mmHg, base excess (BE) −2.2, HCO₃⁻ 22.6 mmol/L, K⁺ 5.0 mmol/L, Na⁺ 135 mmol/L, Cl⁻ 105 mmol/L, glucose 8.0 mmol/L, PaO₂ 273 mmHg, hematocrit 36.1%, hemoglobin 118 g/L, SO₂ 100%, lactate 1.9 mmol/L, Ca^2^⁺ 1.21 mmol/L.

Intermittent phenylephrine was used to maintain blood pressure during the procedure. The operation lasted approximately one hour. At the end of surgery, blood gas analysis had significantly improved: pH 7.404, PaCO₂ 38.5 mmHg, BE −0.5, HCO₃⁻ 24 mmol/L, K⁺ 4.6 mmol/L, Na⁺ 138 mmol/L, Cl⁻ 109 mmol/L, glucose 5.7 mmol/L, PaO₂ 141 mmHg, hematocrit 33.9%, hemoglobin 111 g/L, SO₂ 100%, lactate 0.7 mmol/L, Ca^2^⁺ 1.05 mmol/L. The infant was transferred intubated to the cardiac intensive care unit (CCU). Hemodynamic parameters and resuscitation timeline are summarized in Table 1.

**Table 1:** Hemodynamic parameters and resuscitation timeline.

Time Point	HR (bpm)	BP (mmHg)	ETCO₂ (mmHg)	SpO₂ (%)	Events/Interventions
Baseline (pre-induction)	115	90/55	—	100	Atropine 0.1 mg + Midazolam 0.25 mg + Cisatracurium 1 mg + Propofol 5 mg + Fentanyl 25*μ*g
Post-intubation (∼1 min)	127 → 95 → 60	67/55 → ↓	30	100	HR dropped to 60 within 2 min; Atropine 0.05 mg IV → no response
After Epinephrine (5μg IV)	60 → 130	85/66	30 → 70	100	Epinephrine 5μg (1μg/kg) IV bolus; facial congestion resolved; ETCO₂ surge temporally associated with restored RV forward flow

BP, blood pressure; ETCO_2_, End-Tidal Carbon Dioxide; SpO_2_, Pulse Oxygen Saturation; IV, Intravenous; bpm, Beats Per Minute.

Upon CCU admission, laboratory tests revealed BNP 639.58 pg/mL and troponin 79.14 ng/L. These markedly elevated biomarkers were consistent with acute myocardial strain and right ventricular wall stress, corroborating the intraoperative event. Chest x-ray showed bilateral pulmonary inflammation and consolidation in the right middle and lower lung fields. Diagnoses of heart failure, myocardial injury, and pulmonary infection were made. The infant received milrinone at 0.5 μg/kg/min, vitamin C for myocardial protection, cephalosporin antibiotics, and nebulization therapy. Six hours after CCU admission, the infant showed signs of adequate spontaneous breathing (regular respiratory effort, stable SpO₂ on minimal support) and was successfully extubated without respiratory distress. After two days of antibiotic therapy, pneumonia improved, and the patient was transferred back to the general ward, with discharge on postoperative day 7 in good condition. At one-month follow-up, the infant remained asymptomatic with stable echocardiographic parameters.

## 3 Discussion and conclusion

The case illustrates a prototypical scenario of acute right RVD during anesthetic induction in an infant with a “large RV, small LV” hemodynamic phenotype. **Crucially, the management was imperfect, and these imperfections are precisely what make this case instructive.** We analyze this event through three questions directly relevant to the practicing anesthesiologist: (1) How to recognize acute RVD in real time? (2) Why did this happen? and (3) What should we do differently?

### 3.1 Clinical recognition of acute RVD

The sequence of events in this case—progressive bradycardia, hypotension, facial venous congestion, and a paradoxical ETCO₂ surge—is consistent with a recognizable clinical pattern of acute RVD.

**First, anatomical substrate:** the patient had residual post-repair RVOT stenosis (diameter 7.3 mm) with a tricuspid regurgitation pressure gradient of 58 mmHg (estimated RVSP ≈ 63 mmHg, pulmonary artery systolic pressure cannot be predicted in presence of residual RVOT obstruction) and concurrent pulmonary stenosis (V = 3.3 m/s, estimated gradient ≈ 44 mmHg), indicating chronic RV pressure overload. Echocardiography demonstrated RV enlargement, LV compression, and marked leftward septal deviation (6). Notably, early postoperative intracardiac pressure measurement showed an RVSP/LVSP ratio of 0.4 (34/80 mmHg), indicating that the RV pressure load had not yet reached systemic levels. However, this measurement was obtained under sedation, positive-pressure ventilation, and high-dose vasoactive support, and echocardiography at one month revealed an RVSP of approximately 63 mmHg with marked RV enlargement and LV compression. Carmosino et al. (7) identified baseline suprasystemic pulmonary arterial hypertension(PAH) (mean PAP > 100% of mean systemic BP) as an independent predictor of major perioperative complications in children with PAH (OR = 8.1, *P* = 0.02). This is analogous to our case: chronic RV pressure overload and PAH share a common pathophysiological basis—exhaustion of RV compensatory reserve—which predisposes the patient to acute decompensation under the additional insults of anesthetic induction and positive-pressure ventilation.
**Second, clinical manifestations:** the acute onset of bradycardia, hypotension, and facial venous congestion immediately after intubation was consistent with systemic venous congestion and acute reduction in RV forward flow. Progressive bradycardia with hypotension is a characteristic feature of acute RVD, reflecting a precipitous drop in RV output and severe LV underfilling. Facial cyanosis with a congestive quality indicated elevated systemic venous pressure and impaired venous return from the superior vena cava territory (5, 8). The subsequent rise in ETCO₂ to 70 mmHg following epinephrine administration was temporally associated with restoration of effective pulmonary blood flow and cardiac output and may represent a useful clinical marker of improved RV forward flow.
**Third, laboratory evidence:** Postoperative elevation of BNP (639.58 pg/mL) and troponin (79.14 ng/L) supported the presence of significant myocardial strain and injury following the event. Elevated BNP/NT-proBNP has been shown to have prognostic value in right heart failure associated with pulmonary hypertension, and troponin elevation correlates with RVD and increased mortality (6); However, these biomarkers remain surrogate markers rather than direct indicators of right heart failure. In this case, the troponin rise was most reasonably interpreted as reflecting RV myocardial ischemia secondary to reduced right coronary artery perfusion, an inference supported by the patient's known severe RV pathology and the clinical trajectory.
**Fourth, therapeutic response:** The lack of response to atropine and prompt improvement following epinephrine administration are difficult to reconcile with a vagal reflex—which typically occurs during laryngoscope placement and responds to atropine—and are more consistent with RV pump failure complicated by myocardial ischemia as the underlying mechanism.

### 3.2 Differential diagnosis

#### 3.2.1 Anesthetic-induced cardiovascular depression

Although propofol, opioids, and volatile anesthetics have dose-dependent negative inotropic and vasodilatory effects, the regimen here was deliberately minimized: propofol 1 mg/kg (vs. 2–2.5 mg/kg), fentanyl 5 μg/kg, and sevoflurane 2%. These doses are unlikely to cause profound depression in patients without pre-existing cardiac disease. Nevertheless, the patient developed severe bradycardia and hypotension immediately after intubation—a degree of collapse not explained by anesthetics alone. Rather, a right ventricle already under chronic pressure overload decompensated under the combined effects of anesthesia, positive-pressure ventilation, and loss of spontaneous effort, with anesthetics serving more as a precipitant than the primary cause. This is supported by the prompt hemodynamic response to epinephrine.

#### 3.2.2 Acute RVOT obstruction

This warrants consideration given the residual RVOT stenosis (7.3 mm, estimated gradient 44 mmHg). A sudden increase in PVR could theoretically exacerbate the fixed stenosis. However, the absence of pre-event desaturation and rapid response to epinephrine make this unlikely as the primary mechanism. The stenosis more likely served as an anatomical predisposition.

#### 3.2.3 Ventilation-related causes

Mechanical ventilation increases intrathoracic pressure, which may impede venous return and reduce RV preload. In a patient with chronic RV pressure overload, this could critically compromise RV output. However, facial venous congestion—rather than pallor or hypovolemia—argues against isolated preload reduction and supports RV pump failure. Pneumothorax and endobronchial intubation were ruled out by clear, symmetrical breath sounds, adequate chest expansion, normal airway pressures, and excellent oxygenation.

#### 3.2.4 Anaphylaxis

Drug-induced anaphylaxis can cause hypotension and facial flushing. This was ruled out by the absence of skin rash, bronchospasm, facial edema, or wheeze.

### 3.3 Pathophysiological mechanisms: a two-phase model

The acute decompensation in this case can be understood as the synergistic effect of two sequential insults to an RV already operating near the limit of its Frank–Starling curve (6). Figure 1 illustrates this two-phase model, which we believe has direct teaching value.

**Figure 1:**
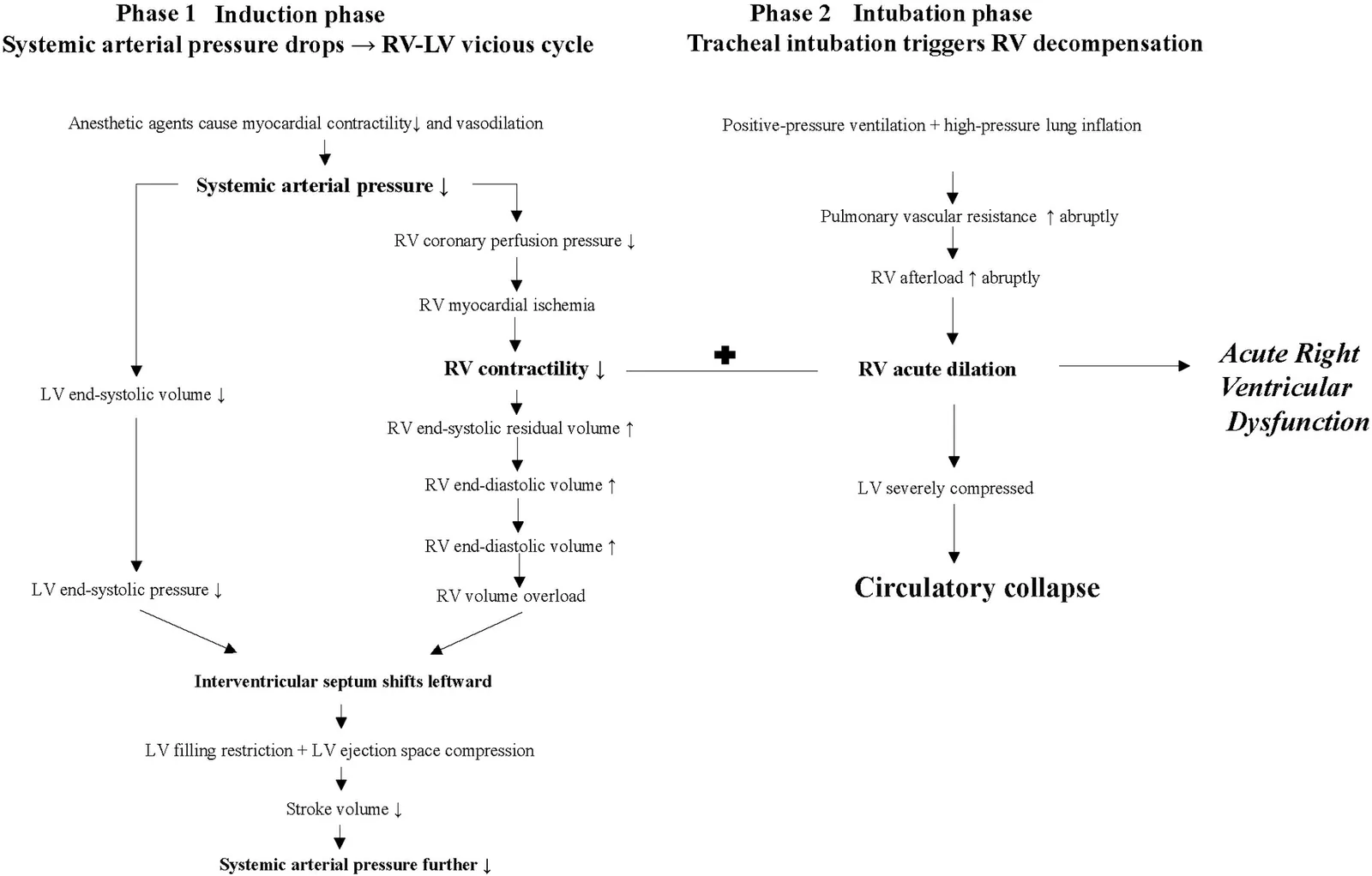
Two-phase model of acute right heart failure and circulatory collapse in an infant with a “large RV, small LV” phenotype. Phase 1: Anesthetic-induced hypotension (propofol + fentanyl + sevoflurane) reduced systolic BP by ∼25% (90→67 mmHg), decreasing RV coronary perfusion and LV end-systolic pressure. This triggered a vicious cycle: ↓BP → ↓RV coronary perfusion → ↓RV contractility → ↑RV dilation → ↑septal shift → ↓LV filling → ↓cardiac output → ↓BP. Phase 2: Positive-pressure ventilation increased PVR via alveolar overdistension and elevated intrathoracic pressure, reducing venous return and RV preload. In a patient with RVOT stenosis, this added afterload exhausted RV reserve, causing acute dilation, worsening tricuspid regurgitation, and critical LV compression. The “final straw” was the combination of both insults—highlighting that risk is cumulative, not event-based. RV, right ventricle.

#### 3.3.1 Phase 1: anesthetic-induced systemic hypotension

Propofol (1 mg/kg), combined with fentanyl and sevoflurane, reduced systolic blood pressure by ∼25% (from 90 to 67 mmHg). This drop had two immediate effects: (a) right coronary perfusion pressure fell, compromising RV oxygen supply in a pressure-overloaded ventricle; and (b) LV end-systolic pressure fell, making the septum more susceptible to leftward displacement by the dilated RV. The vulnerability of the pressure-overloaded RV to even modest reductions in systemic pressure is well established. Under normal conditions, right coronary perfusion occurs during both systole and diastole. However, in the setting of acute or chronic severe pulmonary hypertension, systolic coronary perfusion is reduced or abolished, and diastolic perfusion becomes limited, which critically diminishes the metabolic reserve of the right ventricle (9). Consequently, a fall in systemic systolic pressure directly impairs right coronary perfusion, particularly in a ventricle already operating near the limit of its compensatory capacity. A vicious cycle ensued: lower systemic pressure → lower RV coronary perfusion → worse RV contractility → greater RV dilation → more septal shift → further LV compression → further fall in stroke volume and systemic pressure.

#### 3.3.2 Phase 2: post-intubation positive-pressure ventilation

Lung inflation abruptly increased PVR via two mechanisms: (a) alveolar overdistension compressed intra-alveolar vessels; and (b) elevated intrathoracic pressure reduced venous return and RV preload. In a patient with pre-existing RVOT stenosis, this added afterload exhausted the RV's compensatory reserve, triggering acute dilation, worsening tricuspid regurgitation, and critical LV compression. The “final straw” was not induction or ventilation alone, but their combination—highlighting that risk is cumulative, not event-based.

The clinical trajectory observed in this case—rapid hypotension with delayed hypoxemia in the absence of an intracardiac shunt—closely aligns with the “Phenotype B (slow hypoxemia, fast hypotension)” framework proposed by Evers et al. (10) in the context of pediatric pulmonary hypertension. According to this paradigm, when right ventricular–vascular uncoupling occurs without an atrial-level communication, the dilated RV compresses the LV chamber, leading to an abrupt fall in LV preload and systemic cardiac output, while arterial hypoxemia emerges only later as mixed venous oxygen saturation declines. Our case provides a real-time clinical validation of this framework and extends it by documenting the temporal sequence of hemodynamic and capnography changes during anesthetic induction in an infant with a “large RV, small LV” phenotype.

### 3.4 Implications for perioperative management

#### 3.4.1 Preoperative assessment

Whenever feasible, a qualified echocardiographic assessment should be performed as close as possible to the planned general anesthesia, ideally on the day preceding surgery. The purpose of this assessment is to delineate the patient's current cardiac anatomy and functional status, with particular emphasis on identifying and characterizing relevant abnormalities such as right ventricular dysfunction or right heart strain. Currently, typical echocardiographic parameters employed for the evaluation of right ventricular function include tricuspid annular plane systolic excursion (TAPSE), right ventricular fractional area change, tricuspid annular systolic velocity determined by tissue Doppler imaging, and right ventricular longitudinal strain. In addition, pulmonary artery systolic pressure estimated from the tricuspid regurgitation jet, the left ventricular eccentricity index, and the D-shaped sign reflecting septal flattening provide complementary information regarding right ventricular afterload, ventricular interdependence, and right ventricular pressure or volume overload, respectively (6, 9). In addition, the POQI 9 RV Risk Score may also be used for preoperative assessment (11). In the present case, the patient's clinical presentation was relatively stable, however, the hemodynamic profile indicated high risk, including RV enlargement with LV compression, residual right ventricular outflow tract stenosis, an estimated RVSP of approximately 63 mmHg, and a BNP of 639.58 pg/mL. This discrepancy between clinical appearance and hemodynamic risk further underscores the value of structured preoperative assessment in infants at risk for RVD.

#### 3.4.2 Hemodynamic support during induction

The most important lesson from this case is that systemic systolic blood pressure is not merely a perfusion marker, but also a therapeutic target in its own right. Maintaining systemic pressure serves three essential functions: (a) preserving right coronary perfusion pressure—critical because RV coronary flow continues throughout the cardiac cycle; (b) providing a “counterforce” against leftward septal shift, maintaining LV geometry; and (c) sustaining LV preload through effective RV ejection. These principles suggest that avoiding clinically significant systemic hypotension and maintaining adequate systemic perfusion pressure are important goals in this population. However, it should be acknowledged that achieving this goal requires a comprehensive approach that integrates preload optimization, RV contractility support, RV afterload reduction, oxygenation, ventilation, acid-base management, and heart rate control—all of which interact to determine overall hemodynamic stability.

Any systemic hypotension should be aggressively treated with judicious fluids and vasoactive medications to avoid the downward spiral of hypotension and RV ischemia. Given the high risk of acute hemodynamic deterioration during induction and initiation of positive-pressure ventilation, vasoactive support should be immediately available and, in selected high-risk patients, considered prophylactically (4, 6).In this case, a low-dose vasoactive infusion (e.g., epinephrine, norepinephrine, or vasopressin) initiated before or simultaneously with induction might have helped maintain systemic arterial pressure and right ventricular coronary perfusion, rather than waiting for hypotension to develop before initiating rescue therapy. This consideration is particularly relevant to the “large RV, small LV” phenotype with a chronically pressure-overloaded right ventricle, in whom even a modest reduction in systemic vascular resistance can rapidly trigger decreased right coronary perfusion, impaired RV contractility, and leftward septal shift. In patients with severe pulmonary hypertension, the availability and continuation of inhaled pulmonary vasodilator therapy (e.g., inhaled nitric oxide) should also be considered.

With regard to drug selection, inotropes such as epinephrine or dobutamine increase RV contractility but may induce tachycardia, which can impair diastolic filling and coronary perfusion; vasopressor therapy with norepinephrine, vasopressin, or terlipressin may increase SVR, reduce leftward septal shift, and improve tissue perfusion. Among these, vasopressin is not believed to increase PVR, whereas norepinephrine and epinephrine can increase PVR at high doses, and phenylephrine may also raise PVR. Accordingly, with respect to PVR effects, vasopressin and norepinephrine appear preferable to phenylephrine. Ideally, norepinephrine or a more afterload-neutral agent should be considered when clinically feasible, administered as a continuous low-dose infusion to maintain systemic pressure throughout induction (6).

Milrinone and levosimendan are inodilators that reduce afterload of both the left and right ventricle and may therefore cause or aggravate arterial hypotension. They are not vasopressors and should not be used as first-line agents in the setting of acute systemic hypotension. Rather, both agents may be useful after initial hemodynamic stabilization, particularly in acute or decompensated right ventricular dysfunction, when reduction of ventricular afterload and improvement of contractility are desired. In the present case, milrinone was used in this manner in the cardiac intensive care setting after adequate systemic perfusion had been established (6).

In this case, however, phenylephrine was used intraoperatively as the primary vasopressor, and no continuous vasoactive infusion was established before induction. Although phenylephrine increases SVR and can support blood pressure, it also raises PVR, which may increase right ventricular afterload—a particular concern in the setting of pre-existing RVD. The use of phenylephrine in this instance, and the absence of prophylactic low-dose vasoactive support during induction, therefore represent limitations of our intraoperative management. Diluted epinephrine (1 μg/mL or 10 μg/mL) should be prepared in advance for rescue in bradycardia or circulatory collapse. Bradyarrhythmia associated with RVD usually signify a preterminal event or severe ischemia of the conduction system and should be treated immediately.

#### 3.4.3 Induction agent selection

Propofol is usually not recommended as the primary induction agent because of its negative inotropic and vasodilatory properties (3, 5), even low-dose (1 mg/kg) may precipitate severe hypotension in these patients. Etomidate and ketamine are commonly regarded as preferred induction agents for patients with pulmonary hypertension. In contrast to propofol, ketamine confers advantages attributable to its sympathomimetic properties, which help preserve systolic blood pressure during induction in patients vulnerable to hypotension (9), its intrinsic analgesic activity further confers an opioid-sparing effect, potentially mitigating the risk of opioid-related respiratory depression. A retrospective cohort study by Williams et al. (12) specifically evaluated the safety of ketamine for general anesthesia in children with PAH. In this cohort, ketamine—whether administered as the sole anesthetic agent or in combination with propofol or volatile anesthetics—was not associated with an increased incidence of perioperative complications. Consequently, for patients at risk of RVD, ketamine may offer advantages over propofol, although comparative data with etomidate remain limited.

#### 3.4.4 Optimization of preload

Preload should be carefully balanced to ensure adequate cardiac output while minimizing RV overdistension. In a right ventricle with pre-existing pressure overload and limited compensatory reserve, volume overload may precipitate RV dilation, which can shift the interventricular septum towards the LV and potentially compromise LV stroke volume through ventricular interdependence. Both hypovolemia and excessive volume loading may therefore be detrimental. An adequate and carefully assessed intravascular volume status should be established before induction when clinically feasible, intraoperative fluid management may be guided by hemodynamic response, with small aliquots considered when preload appears inadequate and fluid restriction or diuresis considered when signs of venous congestion predominate. A CVP target of 8–12 mmHg has been suggested, and preserved mixed venous oxygen saturation (SvO₂ > 65%) with normal lactate may provide reassurance that cardiac output is adequate (11). When volume overload is present, diuretics are generally first-line. Loop diuretics and sodium excretion can be used to achieve optimal preload, and ultrafiltration may be considered if diuresis is inadequate or renal replacement therapy is indicated (13). In our patient, the markedly enlarged RV with a compressed small LV cavity suggested that excessive fluid administration could have further worsened septal shift and LV filling, thus a more restrictive volume strategy, guided by echocardiographic assessment of ventricular interaction, might therefore have been preferable.

#### 3.4.5 Ventilation strategy: Lung-protective but RV-friendly mechanical ventilation

This case highlights the potential hemodynamic consequences of lung inflation after intubation in a patient with pre-existing RVD. Based on this single-case experience, we offer the following preliminary considerations for ventilation management in similar high-risk infants.

Excessive lung inflation should be avoided. Mechanical ventilation can increase intrathoracic pressure, thereby impede venous return and reduce RV preload, for a right ventricle with pre-existing pressure overload and limited compensatory reserve, even a modest reduction in preload may markedly decrease stroke volume, making this effect particularly poorly tolerated (9). In addition, positive-pressure ventilation may increase PVR, which can occur both at high lung volumes, when intra-alveolar vessels become compressed, and at low lung volumes, when hypercarbia and hypoxic pulmonary vasoconstriction supervene. Accordingly, ventilatory strategies should maintain lung volume within an optimal range, avoiding both overdistension and atelectasis.

Pressure-controlled ventilation may be considered, with driving pressure limited to minimize transpulmonary pressure and its adverse effects on RV afterload. Positive End-Expiratory Pressure (PEEP) should be individualized and titrated to hemodynamic response, avoiding impairment of venous return and RV output. The lowest effective PEEP that maintains adequate oxygenation (suggested target 5–10 cmH₂O) seems reasonable, and routine use of a fixed PEEP range without physiological justification appears inadvisable. Where available, general management parameters may include pH ≥7.4, plateau pressures <27–30 cmH₂O, tidal volumes of 6–8 mL/kg predicted body weight, and SaO₂ > 92%, all aimed at minimizing intrathoracic pressure–induced PVR elevation while maintaining adequate oxygenation and ventilation (9, 11).

Respiratory rate should be adjusted to maintain ETCO₂ within a range that avoids hypercapnia, which directly elevates PVR. Continuous ETCO₂ monitoring is recommended, an abrupt change should raise concern for altered cardiac output, rather than being attributed solely to ventilator or airway issues (14). Hypercapnia, acidosis, hypothermia, and noxious stimuli all increase PVR and are recognized causes of acute pulmonary hypertensive crisis under anesthesia (9).

#### 3.4.6 Prepared vasoactive rescue——the role of epinephrine

This case demonstrated a prompt response to 5 μg (1 μg/kg) of epinephrine. Epinephrine exerts both *β*₁- and *α*₁-adrenergic effects: (1) *β*₁-mediated inotropy enhances RV contractility, promoting forward flow and relieving LV compression; (2) *α*₁-mediated vasoconstriction raises systemic pressure, relieves LV compression, and improves right coronary perfusion. In pediatric patients, epinephrine is a well-established agent for increasing oxygen delivery in RHF.

Of particular note is the concern that epinephrine should be used cautiously in patients with RVOT stenosis due to the potential for dynamic obstruction from increased inotropy (15). However, in this case the stenosis was a **fixed**, postoperative residual anatomical narrowing rather than a dynamic, contraction-induced obstruction. This distinction is clinically critical: in fixed stenosis with severe bradycardia, low-dose epinephrine (1 μg/kg) was effective in this case and may be considered as a rescue option. Echocardiographic assessment of RVOT morphology—specifically, whether the narrowing is fixed (anatomical) or dynamic (systolic variation)—is essential for this distinction and should be documented in the preoperative planning. Nevertheless, we acknowledge that this recommendation is derived from a single case and should not be generalized as an established standard dose specifically for RV failure, further experience is needed to validate its safety and efficacy.

#### 3.4.7 Preparation for a pulmonary hypertensive crisis

If a pulmonary hypertensive crisis occurs, immediate resuscitation should aim to rapidly reduce PVR, maintain SVR and coronary perfusion, and support RV function. Immediate measures include 100% oxygen, mild hyperventilation, a selective pulmonary vasodilator (e.g., iNO), ventricular support, systemic alkalinization, and deepening anesthesia if noxious stimuli are a trigger. With respect to volume, RV function is preload-dependent in pediatric PH, a fluid bolus may be helpful if hypovolemia is suspected, but may worsen decompensated RV failure, so volume therapy should be guided by hemodynamic response.

The choice of pulmonary vasodilator warrants particular consideration. Efficacy may be reduced in long-standing PAH with non-reactive vasculature, and iNO is generally preferred in mechanically ventilated patients because it rapidly and selectively reduces PVR without significantly lowering SVR, other agents such as sildenafil, milrinone, and prostanoids also lower SVR and may worsen hypotension and coronary perfusion. As discussed above, vasopressin may be useful in this setting because it increases SVR while decreasing PVR, whereas phenylephrine and norepinephrine increase PVR. The impact of afterload manipulation in the setting of a post-tricuspid shunt must be considered, and echocardiography during resuscitation is strongly recommended (9).

## 3.5 Communication with the parents

The risks of anesthesia and surgery were discussed with the parents preoperatively. When the critical event occurred, the parents were informed intraoperatively and told that the patient would be transferred to the cardiac intensive care unit postoperatively. The situation and its implications were explained to them, and close communication was maintained thereafter. They were also informed that this complication could still have occurred despite optimal preparation and management.

## 3.6 Limitations

Several limitations should be acknowledged. First, this is a single case report, and the findings may not be generalizable. Second, invasive hemodynamic monitoring was not available. Third, an echocardiogram was not performed immediately prior to the orthopedic procedure, and multidisciplinary consultation (including pediatric cardiology and cardiac surgery) was not formally sought before surgery—both of which would be advisable in similar high-risk patients. Last, preoperative BNP and troponin levels were not measured prior to the orthopedic procedure, as the patient was asymptomatic and the procedure was considered short and minor, which also represents a limitation.

In conclusion, this single case illustrates a prototypical sequence of acute RV decompensation during induction in an infant with repaired truncus arteriosus and a “large RV, small LV” phenotype. Our observations suggest that: (1) multiple factors—pre-existing RVD, induction anesthetics, positive-pressure ventilation, and lung overinflation—may have collectively contributed; (2) maintaining systemic systolic pressure appeared important for RV perfusion and septal stability; (3) excessive lung inflation should be avoided; (4) low-dose epinephrine was effective as a rescue agent in this setting of fixed RVOT stenosis with bradycardia. These findings are preliminary and derived from a single case, they should serve as clinical reminders rather than practice guidelines, and require further validation.

## Data Availability

The original contributions presented in the study are included in the article/Supplementary Material, further inquiries can be directed to the corresponding author.
